# Investigation of Microwave Ablation Process in Sweet Potatoes as Substitute Liver

**DOI:** 10.3390/s21113894

**Published:** 2021-06-04

**Authors:** Muhammad Saad Khan, Michael Hawlitzki, Shadan Mofrad Taheri, Georg Rose, Bernd Schweizer, Andreas Brensing

**Affiliations:** 1 Department of Engineering, RheinMain University of Applied Sciences, 65428 Ruesselsheim, Germany; michael@hawlitzki.com (M.H.); shadan.taheri@hotmail.com (S.M.T.); bernd.schweizer@hs-rm.de (B.S.); andreas.brensing@hs-rm.de (A.B.); 2 Institute of Medical Engineering and Research Campus STIMULATE, Otto Von Guericke University, 39106 Magdeburg, Germany; georg.rose@ovgu.de

**Keywords:** liver, microwave ablation, sweet potato, temperature monitoring

## Abstract

The microwave ablation technique to destroy cancer tissues in liver is practiced clinically and is the subject of ongoing research, e.g., ablation monitoring. For studies, liver tissue from cattle or pigs is often used as a substitute material. In this work, sweet potato is presented as an alternative material for microwave ablation experiments in liver due to similar material properties. Sweet potatoes as a substitute for liver have the advantages of better handling, easy procurement and stable material properties over time for microwave ablation experiments. The dielectric constant and electrical conductivity of sweet potato are characterized for temperature variation with the help of high-temperature dielectric probe. Furthermore, a test setup is presented for microwave ablation experiments in which a bowtie slot antenna matched to sweet potato is placed on its surface to directly receive the microwave power from a self-developed microwave applicator inserted into a sweet potato 4 cm below the surface antenna. A high-power source was used to excite the microwave powers up to 80 W and a spectrum analyzer was used to measure the signal received by the surface antenna. The experiments were performed in an anechoic chamber for safety reasons. Power at 50 W and 80 W was stimulated for a maximum of 600 s at the 2.45 GHz ISM band in different sweet potato experiments. A correlation is found between the power received by the surface antenna and rise of temperature inside sweet potato; relative received power drops from 1 at 76 ${}^{\circ }$C to 0.6 at 88 ${}^{\circ }$C (max. temperature) represents a 40% relative change in a 50 W microwave ablation experiment. The received power envelope at the surface antenna is between 10 mW and 32 mW during 50 W microwave ablation. Other important results for 10 min, 80 W microwave ablation include: a maximum ablation zone short axis diameter of 4.5 cm and a maximum ablation temperature reached at 99 ${}^{\circ }$C, 3 mm away from the applicator’s slot. The results are compared with the state of the art in microwave ablation in animal liver. The dielectric constant and electrical conductivity evolution of sweet potato with rising temperature is comparable to animal liver in 50–60 ${}^{\circ }$C range. The reflection loss of self-developed applicator in sweet potato is below 15 dB which is equal to reflection loss in liver experiments for 600 s. The temperature rise for the first 90 s in sweet potato is 76 ${}^{\circ }$C as compared to 73 ${}^{\circ }$C in liver with 50 W microwave ablation. Similarly, with 80–75 W microwave ablation, for the first 60 s, the temperature is 98 ${}^{\circ }$C in sweet potato as compared to 100 ${}^{\circ }$C in liver. The ablation zone short-axis diameter after 600 s is 3.3 cm for 50 W microwave ablation in sweet potato as compared to 3.5 cm for 30 W microwave ablation in liver. The reasons for difference in microwave ablation results in sweet potato and animal liver are discussed. This is the first study to directly receive a signal from microwave applicator during a microwave ablation process with the help of a surface antenna. The work can be extended to multiple array antennas for microwave ablation monitoring.

## 1. Introduction

Liver cancer is the seventh most likely and the second most deadly cause of cancers worldwide; in 2018, mortality rate was 93% out of total new cases reported [1], while in another reference, a 20% 5-year relative survival rate was reported [2]. Thus, the early detection and rapid treatment of liver cancer is crucial. Hyperthermia [3] and microwave hyperthermia [4] were proposed as early as 1980 for the treatment of different cancers in the human body. Some recent applications of hyperthermia have been in cardiac ablation [5], in the enhancement of low-density lipoprotein (LDL) transport in arteries [6], and in drug delivery enhancement [7,8,9]. Thermal ablative techniques to destroy liver and other cancer tissue cells using laser, radio frequency (RF) and microwaves has been practiced clinically for the past two decades [10,11]. However, microwave ablation (MWA) outshines the others as an effective treatment due to the advantages it offers of ablating comparatively large tissue in a smaller amount of time as compared to RF and laser ablation [12].

Furthermore, monitoring the growth of the ablation zone during a MWA is a current research problem which is related to the safety of the tissues and organs close to the infected area. Many approaches are being investigated to tackle this problem; some of which are invasive like temperature monitoring using fiber Bragg grating sensors [13,14] and using localized probes at the tip of the microwave applicator [15]. However, invasive approaches give localized information at a point or along a line, insufficient for the temperature mapping of the ablation zone. Additionally, deploying and locating these sensors is also a challenge. Magnetic resonance imaging (MRI)-based thermometry is a non-invasive technique for temperature monitoring during MWA but then the complete intervention has to be performed inside an MRI scanner [16,17]. Hence, microwave imaging using body matched antennas (BMAs) is experimented as an independent non-invasive technique for real-time imaging and monitoring the thermal ablation process [18,19]; however, in [18], an array of ultra-wideband (UWB) antennas is used in a bistatic radar principle to carry out the reconstruction. Another simpler approach for carrying out microwave imaging by using a microwave applicator as a transmit antenna and a surface BMA as a receiving antenna at 2.45 GHz was suggested in [20] by the authors.

For understanding MWA in liver, experimental studies are performed on animal liver to record localized temperature, temperature dispersive material properties and the spread of the ablation zone with ablation time and power. However, the material properties of an ex-vivo liver are highly instable, as with increasing time after the slaughter, the animal liver rapidly loses water and its electrical conductivity also changes. Therefore, there is a need for a substitute material for experimental studies for monitoring of MWA in liver. The primary goal of this work is to present sweet potatoes (SPs) as a substitute material for MWA experiments on liver and the rationale for doing so are the similar material properties of SP and liver. The properties of SPs, as reported in [21], motivated the authors to explore SPs as alternative to liver due to similar dielectric constant and electrical conductivity. Table 1 shows the comparison of dielectric constant, electrical conductivity, water content and the density of liver and SP at room temperature at 2.45 GHz. All the properties are very similar to each other and the maximum difference between any property is 19% as in case of the dielectric constant.

In addition to having similar material properties, there are also other advantages for using SPs instead of animal liver for MWA experiments. Firstly, SPs are easier to handle than animal liver because there is no blood involved during the experiments which needs proper handling. Also from a logistic point of view, a raw animal liver cannot be stored for long even with cooling while SPs are easy to procure. Secondly, the properties of SPs are more stable over time, which in case of a slaughtered animal liver, drastically change due to the loss of moisture content which is 70–80% of the total liver meat volume [23]. Additionally, during an ex-vivo MWA, when the inner part of the liver is ablated, the outer liver does not give the same liver properties as one would expect in an in-vivo environment. On the other hand, in SPs the properties of the outer part remain the same during a MWA experiment.

Furthermore, in this paper, a system for carrying out MWA experiments is presented. This system is useful for measuring temperature development during ablation process, ablation zone short axis diameter (SAD), testing new microwave applicator designs and microwave field distribution for use in a monitoring system with the help of BMAs. A self-developed microwave applicator and a similar bow-tie BMA [20] are used during the course of experiments.

## 2. Materials and Methods

**Figure 1:**
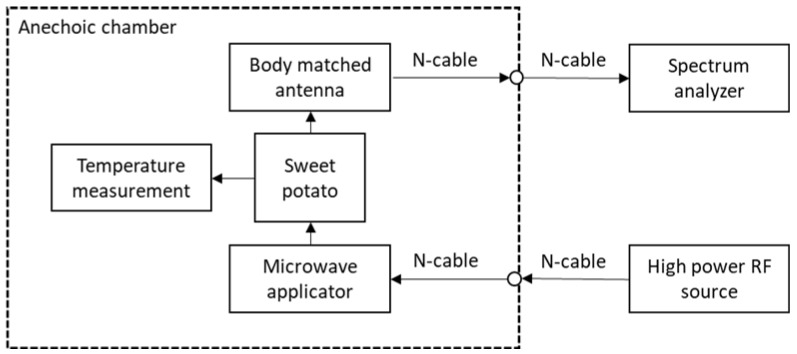
MWA experiment flowchart.

A microwave applicator, BMA, high-power RF source, anechoic chamber, fiber optic temperature sensor, spectrum and vector network analyzers were used for the experimental work. The block diagram of the MWA experiments is shown in Figure 1.

**Figure 2:**
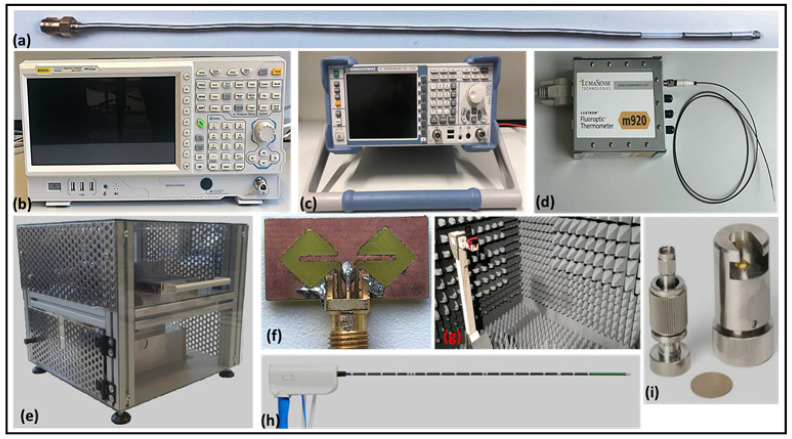
MWA experimental setup: (**a**) self-developed microwave applicator with a single slot; (**b**) spectrum analyzer; (**c**) VNA ; (**d**) LumaSense temperature probe; (**e**) high-power RF source; (**f**) BMA; (**g**) anechoic chamber; (**h**) Covidien Emprint™ Standard Percutaneous Antenna with Thermosphere™ Technology; (**i**) N1501A high-temperature probe kit.

A self-developed microwave applicator with a slot (15 mm) before the tip was used for exciting 50–80 W microwave power in SP at 2.45 GHz. A commercial applicator offered by COVIDIEN [25,26] was also used to benchmark the self-developed applicator. The received antenna used is a similar bowtie-slot microstrip patch BMA with an edge-fed Sub-Miniature version-A (SMA) connection designed for 2.2–2.8 GHz [20]. A Kuhne electronic signal generator (KU SG 2.45–250 A) controlled by a graphical user interface (GUI) was used to excite high power for MWA [27]. The power can be varied from 10 to 250 W, in increments of 10 W. The operational frequency band was from 2.4 to 2.5 GHz, which can be changed in the 0.05 GHz steps. The reflected as well as the excited power can be monitored in the GUI. Since the experiments involve high-power microwave radiations, the microwave applicator inserted into the SP along with the receiving BMA were placed in the anechoic chamber at the HF Lab in RheinMain University of Applied Sciences. For the temperature measurement, a fiber optic temperature sensor of the LumaSense technologies of the m920 series was used [28]. The 3 mm tip of the (STF) probe was sensitive to the temperature changes from 0 to 295 ${}^{\circ }$C with a response time of 0.25 s. The spectrum analyzer from Rigol (RSA3045) with a frequency range from 9 kHz to 4.5 GHz and dynamic range between −50 dBm and +30 dBm was used to record the received antenna power during the experiments [29]. The reflection losses ($S_{11}$, $S_{22}$) of the microwave applicators and the BMA and the insertion loss ($S_{21}$) were measured with a two-port vector network analyzer (VNA) from Rohde and Schwarz (R&S®ZVL13) [30]. The experimental setup discussed above is shown in Figure 2.

**Figure 3:**
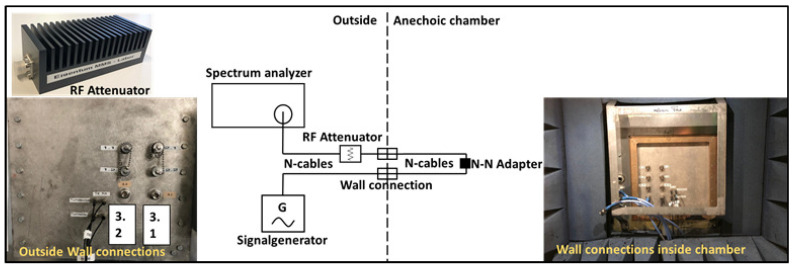
Network loss measurement setup.

The block diagram of the measurement of network loss is shown in Figure 3. To determine the network loss due to N-cables and adapters, a transmission measurement was performed. A signal generator and a spectrum analyzer were connected to the N connections on the wall of anechoic chamber from outside and then two more N cables were connected from inside of the chamber which were short circuited to each other through an N–N female adapter. An RF attenuator of 30 dB was used for the protection of the spectrum analyzer in the received path for supplying powers in excess of +30 dBm which was the max. power rating of the spectrum analyzer. An input power of 10 dBm at 2.45 GHz was supplied from the signal generator and −25.8 dBm power was received at the spectrum analyzer; thus, a total loss of 35.8 dB is calculated, which includes the network loss of 5.8 dB.

**Figure 4:**
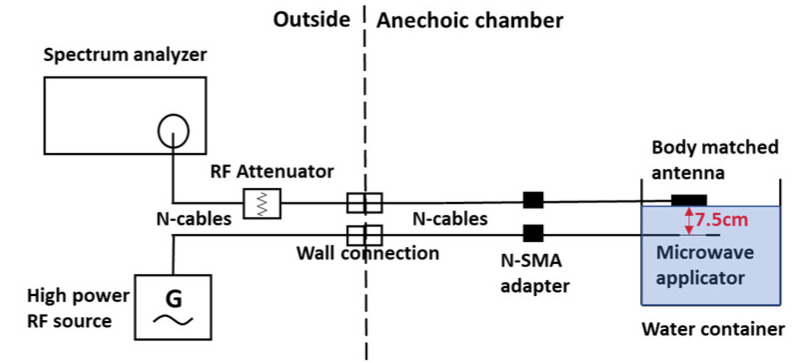
MWA experimental setup testing in water.

The measurement setup for the SP experiments was first tested with double-distilled water as a medium. A water container, BMA placed on the water surface and microwave applicator dipped 7.5 cm (distance between the BMA center and the applicator slot) below the water surface, were placed in the anechoic chamber. The BMA and the applicator were connected to the N connections on the chamber wall. A transmitted power of 47 dBm at 2.45 GHz was supplied and a received signal of −40.6 dBm was measured. The total power loss was 87.6 dB and after subtracting the loss of 35.8 dB due to the attenuator and the network, the attenuation in the double-distilled water was 51.8 dB, which is in good agreement with the simulation value of 51.5 dB. The setup is shown in Figure 4.

**Figure 5:**
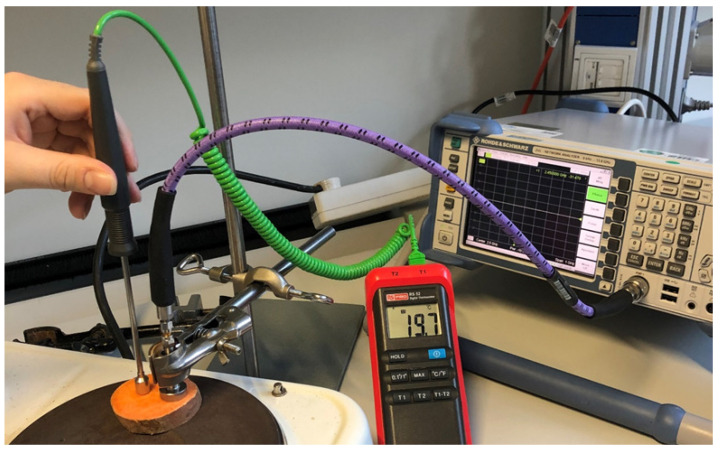
Experimental setup for measuring material properties of SP.

It is important to characterize the material properties of SP with respect to temperature. For this purpose, the dielectric and conductive properties of SP were determined by increasing temperature with a heating plate, digital thermometer, high temperature probe [31] and VNA at 2.45 GHz. The SP was cut into 1 cm-thick slices and placed on the heating plate. The probe was then placed on the surface of the SP as shown in Figure 5.

**Figure A1:**
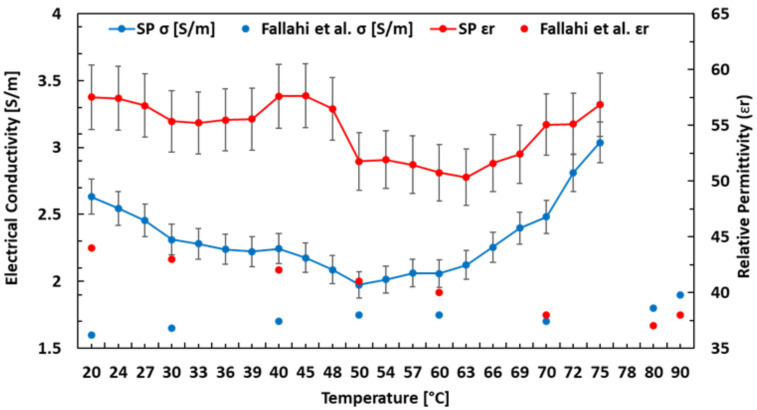
Change in electrical conductivity and dielectric constant of SP and liver with temperature at 2.45 GHz.

The calculated material properties of the SP are shown in Table 2. The electrical conductivity of the SP decreases from 2.6 S/m at room temperature to 2.1 S/m at 60 °C. At temperatures above 60 °C, the σ increases due to the formation of water between the probe and the SP, the $\epsilon_{r}$ shows a similar behavior. The measurement method has an uncertainty of 10%. The evolution of material properties of SP with temperature are compared with the liver below 80 ${}^{\circ }$C as in [32]. The change in the properties show a similar trend between SP and the liver. The comparisons of temperature-dependent $\epsilon_{r}$ and σ of SP and liver are shown in Figure A1.

**Figure 6:**
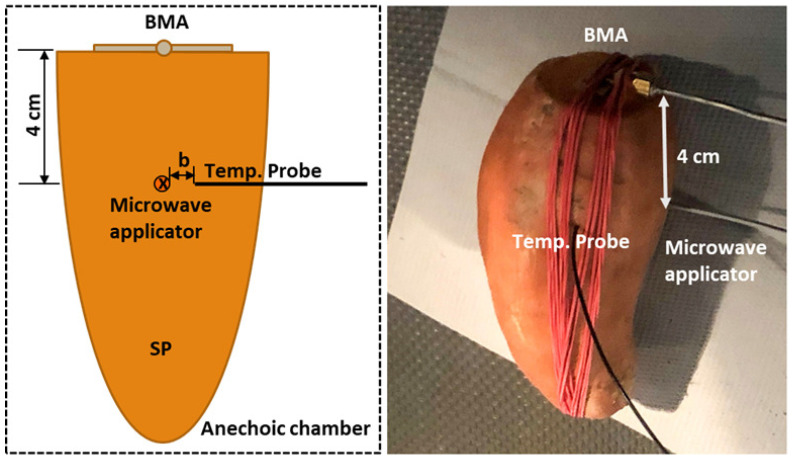
MWA experimental setup for SP.

Figure 6 shows the setup of an MWA experiment of a SP in an anechoic chamber. One end of the SP was cut to place the BMA on the SP surface. The BMA was firmly fixed on the surface with help of rubber bands so that the BMA does not move during the measurements and there is no air gap between the BMA and the surface of SP. The center-to-center distance between the BMA and the applicator’s slot is always 4 cm. The hole for inserting the microwave applicator is marked vertically 4 cm below the SP. The exact position of the applicator and temperature sensor’s hole and the hole depth are determined by using a level ruler.

## 3. Results

**Figure A2:**
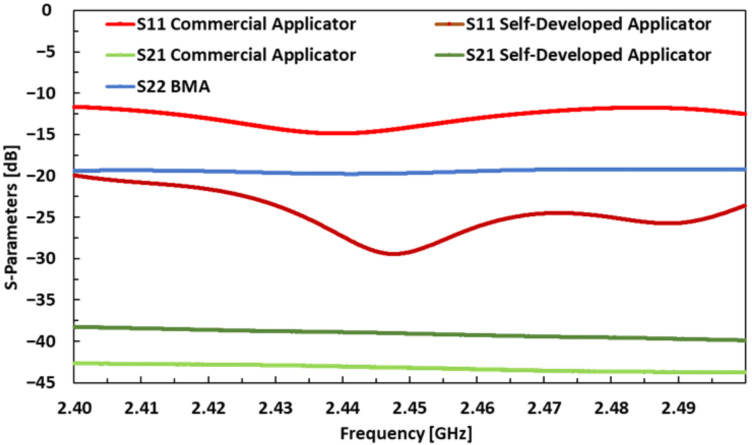
S-parameters measurement of self-developed microwave applicator, commercial applicator and BMA in SP.

The S-parameters of both microwave applicators in SP and the BMA on SP are measured from 2.4 to 2.5 GHz and compared at 2.45 GHz, as shown in Figure A2. The $S_{11}$ (reflection loss of the self-developed applicator) is −29.2 dB, compared to −14.1 dB for the commercial applicator. Then, $S_{22}$ (reflection loss of the BMA) is −19.7 dB which is below the −10 dB threshold. Furthermore, $S_{21}$ (power received at the BMA) is −39.1 dB for the self-developed applicator as compared to −43.2 dB for the commercial applicator.

**Figure A3:**
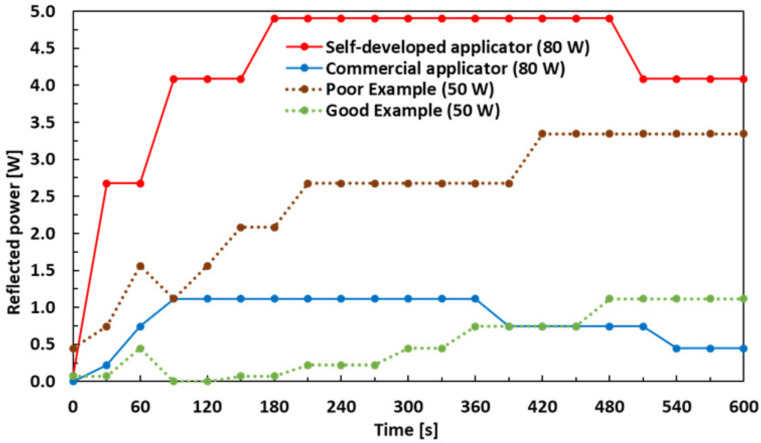
Comparison of reflected powers of commercial and self-developed applicators for 80 W MWA and poor and good examples of self-developed applicator matching for 50 W MWA in SP.

**Figure A5:**
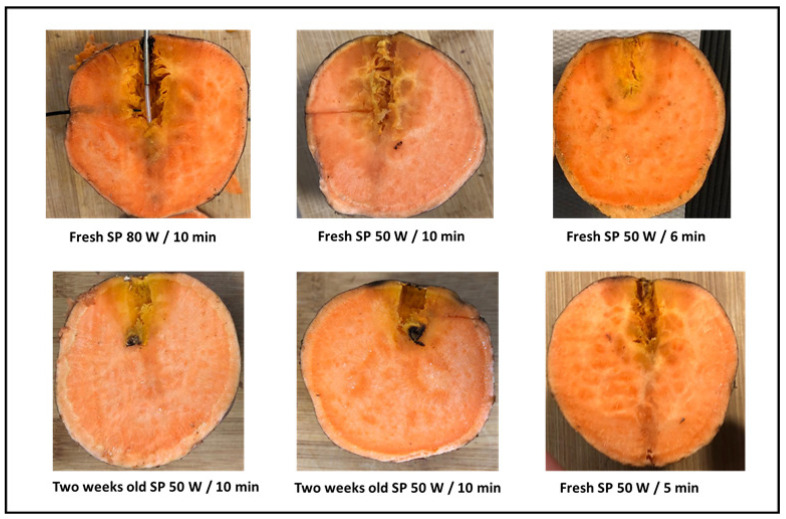
MWA result examples of SP.

The reflected power of the 10 min MWA experiment with the two applicators at 80 W for 2.45 GHz is shown in Figure A3. The self-developed and the commercial applicator show a constant reflected power of 4.905 W and 1.115 W, respectively, for about 5 min. It can be observed that the curve of the reflected power of both applicators develops similarly, and the reflected power of the self-developed applicator is higher than that of the commercial applicator. The change in reflected power with time can be attributed to the applicator miss-matching due to the progressive ablation process. As can be seen in the photo of the cut open SP in Figure A5, a burn hole forms immediately around the applicator at high power (80 W). After the validation and benchmarking of the test equipment, the self-developed applicator and the BMA: received and reflected power during MWA, ablation zone SAD as a function of time and received power as a function of temperature are presented.

**Figure 7:**
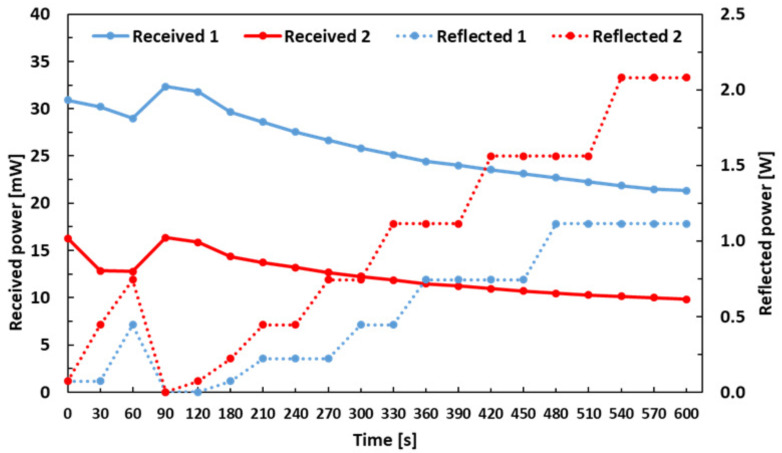
Received and reflected power vs. time for 50 W MWA in SP.

The received and reflected powers at 2.45 GHz during MWA at 50 W for 600 s in SP are shown in Figure 7. The experiment was performed a number of times on different days with the good and poor coupling of microwave power as shown in Figure A3; however, the results presented here, are with the most stable and consistent experimental environment on a same day. In a first measurement (blue curve), the received power drops from 30.9 mW to 29 mW in the first minute. It then rises to 32.4 mW after another 30 s. Afterward, this shows a steady drop from 32.4 mW to 21.3 mW in last 510 s of the measurement. The reflected power in first measurement increases from 0.1 W to 0.4 W in the first minute. At 90 s, no reflected power is measured. After that, the reflected power gradually increases to a maximum value of 2.1 W. Similarly, in the second measurement (red curve), the received power starts at 16.3 mW and drops to 12.8 mW in the first 60 s. After another 30 s, it increases to 16.4 mW and then drops steadily to 9.8 mW after 600 s. The reflected power of the second measurement also increases from 0.1 W to 0.7 W in the first 60 s. No reflected power is measured for 90 s again and afterwards, the reflected power linearly increases from 0 W to 2.1 W until the end of measurement at 600 s.

**Figure 8:**
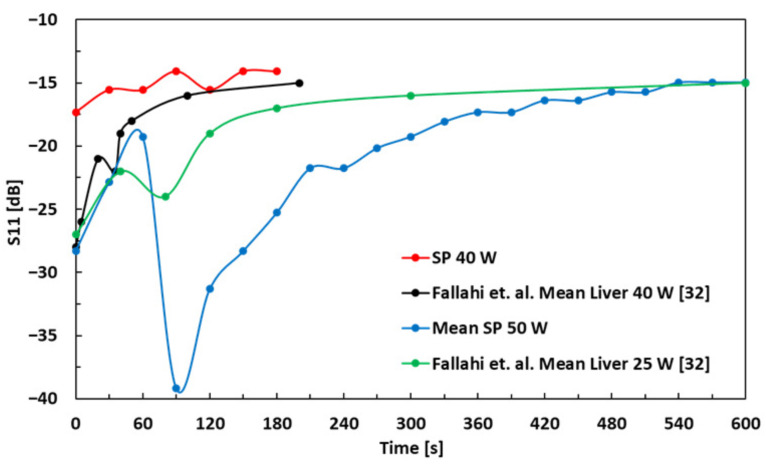
$S_{11}$ as a function of time for 25 W [32], 40 W [32] and 50 W MWA experiments.

The $S_{11}$ (reflection loss) as a function of time for 50 W and 40 W in SP, and 25 W and 40 W in bovine liver, is shown in Figure 8. For the 50 W experiment, the $S_{11}$ of the SP increases from −28.3 dB to −19.2 dB in the first minute. Afterwards, it drops to −39.8 dB at 90 s and then gradually increases to −15 dB at 600 s, which is comparable to 25 W MWA experiment in bovine liver for 600 s, as reported in [32]. The measurement values represented by green curve are taken from Figure 7 in [32]. Similarly, for the 40 W experiment, the $S_{11}$ of the SP increases from −17.3 dB to −14.1 dB in 180 s, which is comparable to the 40 W MWA experiment in bovine liver for 210 s, as reported in [32]. The measurement values represented by black curve are also taken from Figure 7 in [32].

**Figure 9:**
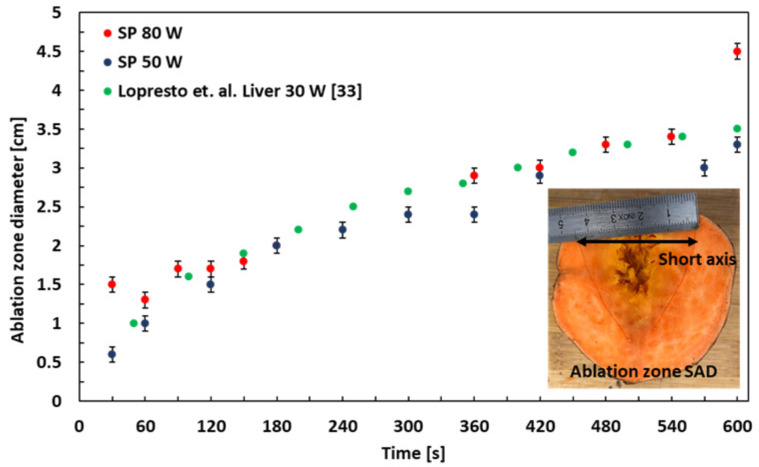
Ablation zone SAD as a function of time for 50 W and 80 W in SP and liver [33].

The ablation zone SAD in SP as a function of time for 50 and 80 W powers at 2.45 GHz is shown in Figure 9 and is also compared with MWA experiment in bovine liver, published in [33]. The ablation zone is elliptical with a short axis perpendicular to the microwave applicator. The measurement values denoted by green dots are taken from Figure 10 in [33], where an MWA experiment in bovine liver using 30 W power was conducted and analyzed, similarly to the method described here. The SP is cut open at the applicator puncture site after each experiment and MWA SAD perpendicular to the applicator channel is noted with an error of ±0.1 cm. The SAD of the ablation zone at 50 W is from 0.6 to 3.3 cm and for 80 W, 1.5 to 4.5 cm.

**Figure 10:**
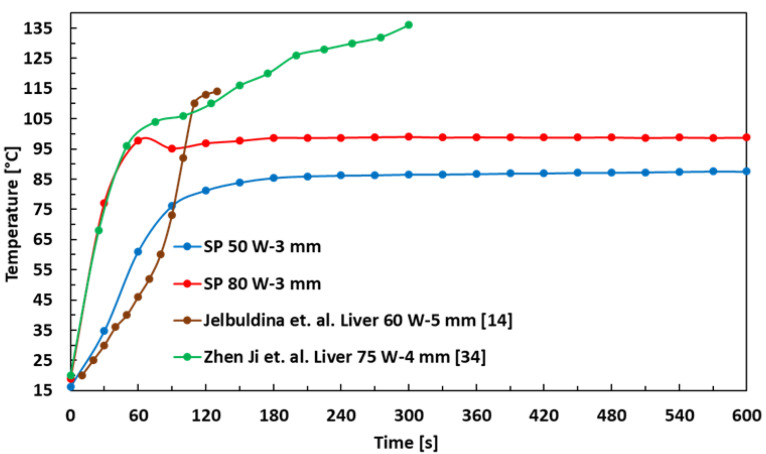
Temperature vs. time in MWA experiments [14,34].

Figure 10 shows the SP MWA temperature as a function of time at 2.45 GHz for 50 and 80 W powers. The temperature measurements of SP are compared with those in MWA experiments in bovine liver at 60 W for 130 s, as reported in [14] and porcine liver at 75 W power for 300 s, as reported in [34]. The measurement values in the brown curve are taken from Figure 6 in [14], corresponding to curve ‘FBG 2.2’ for 5 mm, and similarly, the measurement values in the green curve are taken from Figure 10c in [34], corresponding to curve ‘A’ for 4 mm. The tip of the temperature sensor is 3–4 mm from the microwave applicator slot in both 50 W and 80 W MWA experiments in SP, as shown in the figure inset. The starting temperature of 50 W SP MWA as in the blue curve, increases from 16.2 ${}^{\circ }$C to 85.4 ${}^{\circ }$C after 180 s and remains constant until the end of the measurement. This is compared to [14] as in the brown curve, the values are comparable at 90 ${}^{\circ }$C after which the temperature values of 60 W MWA of liver keeps on increasing until 114 ${}^{\circ }$C at 130 s. Similarly, the starting temperature of 80 W SP MWA as in the red curve, increases from 18.9 ${}^{\circ }$C to 98.7 ${}^{\circ }$C after 180 s and remains constant until the end of the measurement. This is compared to [34] as in the green curve, the values are comparable at 60 ${}^{\circ }$C after which the temperature values of 75 W MWA of liver keep on increasing until 136 ${}^{\circ }$C at 300 s.

**Figure 11:**
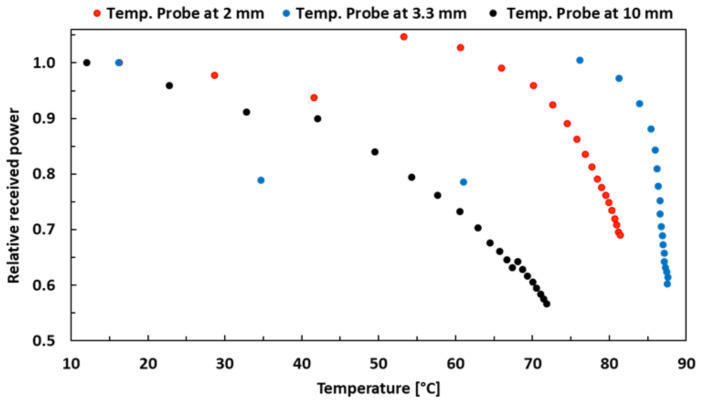
Received power vs. temperature for 50 W MWA in SP.

The relative received power is shown as a function of temperature inside SP for 50 W MWA in Figure 11. Three measurements are taken with the position of the temperature probe at 2 mm, 3.3 mm and 10 mm away from the center of the ablation zone. In all three measurements, it is observed that after a certain temperature, the relative received power starts decreasing with the increase in temperature. In the measurement with the temperature probe at 3.3 mm away from the center of the ablation zone (blue dots), the relative received power drops from 1 at 76 ${}^{\circ }$C to 0.6 at 88 ${}^{\circ }$C, representing a 40% relative change. Therefore, a correlation can be drawn between a rise in temperature and a decrease in received power which could be attributed to more variation in material properties with rising temperature and thus increased losses in SP due to heating. Finally, some pictures of the SP MWA results for different power and time settings are shown in Figure A5. A summary of all quantitative results with minimum and maximum values for different time and temperature settings, for 50 W and 80 W MWA experiments in SP, is shown in Table 3.

## 4. Discussion

Two microwave powers, 50 W and 80 W, are used for MWA in SP. In MWA with 80 W, some burning of the tissue can be observed, as shown in the MWA example of a fresh SP at 80 W/10 min in Figure A5. Therefore, powers above 80 W are not used due to burning effects and also because of the maximum power rating of the self-developed applicator (93 W). However, a comparison is made between 50 W and 80 W MWA experiments in terms of maximum and minimum received powers, reflected powers, ablation zone SADs, time-dependent temperatures and temperature-depended received powers, as illustrated in Table 3. As expected, the maximum reflected power and minimum received power, ablation zone SAD and temperature is more for 80 W MWA than 50 W.

The results were also compared with the state of the art in the MWA of animal liver. The comparison of dielectric constant, electrical conductivity, reflected power, ablation zone SAD temperature as a function of time between SP and animal liver is shown in Table 4. A close agreement of values support SP as an alternative tissue material for MWA experiments in liver. As shown in Table 4 and Figure A1, the $\epsilon_{r}$ and σ of SP and liver are comparable in the 50–60 ${}^{\circ }$C temperature range. However, as the temperature increases, the liver loses water rapidly and thus $\epsilon_{r}$ and σ also decrease sharply but since the water in SP accumulates with increasing temperature, $\epsilon_{r}$ and σ increase above the room temperature value.

The measure of reflection loss of the microwave applicators in liver and SP not only demonstrates an efficient design of the applicator, but also depicts the matching of the applicator to the material itself. Therefore, an almost equal reflection loss for SP and liver is due to the same material characteristics as observed in Table 1. Secondly, as observed in Figure 10 and Table 4, the temperature in SP and animal liver is equal until a point after which the temperature remains constant in SP but keeps on increasing in the MWA in animal liver. A possible explanation for this observation is that in MWA in animal liver, the water loss is rapid due to MWA and also from the open surface of the liver. On the contrary, in case of SP, the skin and outer tissues of the SP stop the water from escaping and the water evaporated by MWA is replaced by the water seeping in from the surrounding tissues of the ablation zone of SP. This phenomena in SP is closer to an MWA of liver in which the loss of water from the liver is not so rapid as compared to the MWA of liver. Lastly, a similar explanation is given for the power difference in the ablation zone SAD comparison in Table 4. It is seen that the ablation zone SAD is slightly bigger with 30 W MWA in the liver than 50 W MWA in SP, which is due to the rapid loss of water in MWA in liver.

This is the first study to directly receive a signal from microwave applicator during a MWA process with the help of a surface BMA. The main target application of this work is the development of an imaging system for temperature monitoring and microwave applicator guidance for MWA. The contrast in tissue dielectric and electrical conductive properties between hot and cold tissue, enable a power change at the surface BMA. The results show that it is possible to recreate MWA process within an SP for simulation studies before trying for actual clinical studies. With the help of the presented test setup, in situ monitoring of the MWA process via surface BMAs can also be done by using the correlation between ablation zone temperature and received power, thereby employing a data processing algorithm for calculating ablation zone dimensions. The test setup can also be used for testing new microwave applicator designs for better matching to the target tissue. A separate work could be carried out for the comparison of temperature development and thermal damage information in SPs with simulation studies. However, the limitation of MWA in SP is that there is no blood perfusion (heat sink) effect in SPs as there is in the MWA of liver and there are no data available for change in the material properties of SP due to tissue burning (above 80 ${}^{\circ }$C).

In conclusion, with help of this work, a correlation could be drawn between the rise in temperature during SP MWA and received power at the surface BMA. The work can be extended to multiple array antennas for the 2D and 3D estimation of the ablation zone. The experimental setup prepared for the purpose of this publication can be used for testing new applicator designs and also in testing other materials for MWA experiments.

## Figures and Tables

**Table 1:** Material properties of liver and SP at 2.45 GHz at room temperature.

Property	Liver	SP
Density	0.95 g/cm${}^{3}$ [22]	1.07 g/cm${}^{3}$ [21]
Water content	80.5% [23]	75% [21]
Dielectric constant ($\epsilon_{r}$)	43 [24]	52 [21]
Electrical conductivity (σ)	1.7 S/m [24]	1.9 S/m [21]

**Table 2:** Temperature-dependent material properties of SP at 2.45 GHz.

Temperature	Electrical Conductivity (σ)	Relative Permittivity ($\epsilon_{r}$)
21 ${}^{\circ }$C	2.6 S/m	57.5
39 ${}^{\circ }$C	2.2 S/m	55.6
60 ${}^{\circ }$C	2.1 S/m	50.7
75 ${}^{\circ }$C	2.8 S/m	55.1

**Table 3:** Summary of all quantitative results for 50 W and 80 W MWA experiments with SP.

Result	50 W (min.)	50 W (max.)	80 W (min.)	80 W (max.)
Reflected power	0 W	2.1 W	0 W	4.9 W
Received power	9.8 mW	32.4 mW	21.6 mW (Figure A4)	30.6 mW (Figure A4)
Ablation zone SAD	0.6 cm (30 s)	3.3 cm (600 s)	1.5 cm (30 s)	4.5 cm (600 s)
Temperature vs. time	16 ${}^{\circ }$C (0 s)	88 ${}^{\circ }$C (600 s)	19 ${}^{\circ }$C (0 s)	99 ${}^{\circ }$C (600 s)
Received power vs. temperature	9.82 mW (88 ${}^{\circ }$C )	32.25 mW (53.5 ${}^{\circ }$C)	-	-

**Table 4:** Comparison between SP and animal liver MWA.

Result	SP	Liver
$\epsilon_{r}$	50.3 (63 ${}^{\circ }$C)	40 (60 ${}^{\circ }$C) [32]
σ (S/m)	1.97 (51 ${}^{\circ }$C)	1.75 (50 ${}^{\circ }$C) [32]
Reflection loss	15 dB (50 W-600 s)	15 dB (25 W-600 s) [32]
Reflection loss	14 dB (40 W-180 s)	15 dB (40 W-210 s) [32]
Temperature	76.1 ${}^{\circ }$C (50 W-90 s-3 mm)	73.0 ${}^{\circ }$C (50 W-90 s-5 mm) [14]
Temperature	97.8 ${}^{\circ }$C (80 W-60 s-3 mm )	100 ${}^{\circ }$C (75 W-60 s-4 mm) [34]
Ablation zone SAD	3.3 cm (50 W-600 s)	3.5 cm (30 W-600 s) [33]

**Figure A4:**
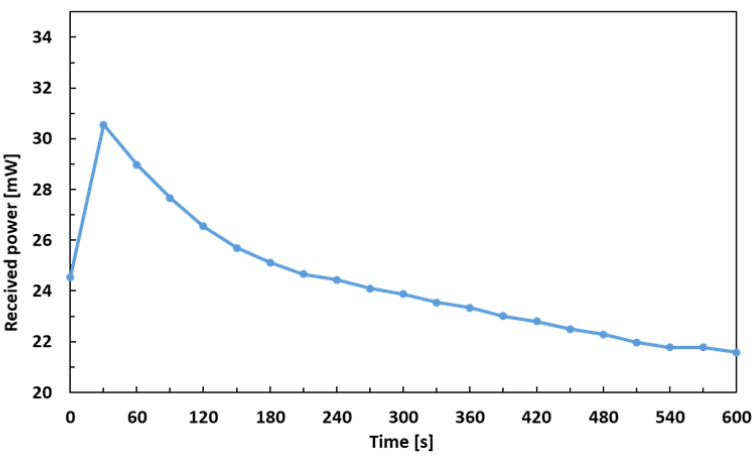
Received power vs. time for 80 W MWA in SP.

## Abbreviations

The following abbreviations are used in this manuscript:

MWA	Microwave Ablation
MRI	Magnetic Resonance Imaging
UWB	Ultra Wide-Band
BMA	Body Matched Antenna
SAD	Short Axis Diameter
SMA	Sub-Miniature Version-A
GUI	Graphical User Interface
VNA	Vector Network Analyzer
RF	Radio Frequency
SP	Sweet Potato
